# 2-[(*E*)-*N*-(Adamantan-1-yl)carbox­imidoyl]-6-eth­oxy­phenol

**DOI:** 10.1107/S1600536812033594

**Published:** 2012-07-28

**Authors:** Muhammad Ashraf Shaheen, M. Nawaz Tahir, Rana Muhammad Irfan, Shahid Iqbal, Mahreen Zaneb

**Affiliations:** a University of Sargodha, Department of Chemistry, Sargodha, Pakistan; b University of Sargodha, Department of Physics, Sargodha, Pakistan

## Abstract

In the title compound, C_19_H_25_NO_2_, the 3-eth­oxy-2-hy­droxy­benzaldehyde group is almost planar (r.m.s. deviation = 0.029 Å). An intra­molecular O—H⋯N hydrogen bond generates an *S*(6) ring. There are no inter­molecular hydrogen bonds.

## Related literature

For a related structure, see: Fernandez *et al.* (2001 ▶).
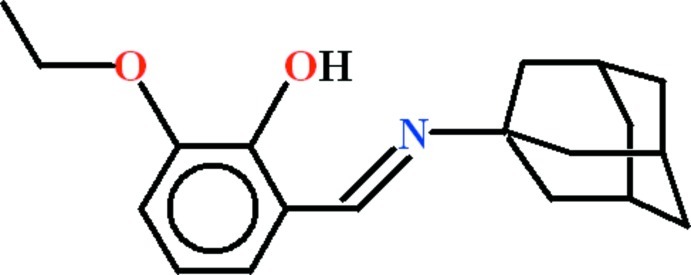


## Experimental

### 

#### Crystal data

C_19_H_25_NO_2_
*M* *_r_* = 299.40
Monoclinic, 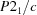

*a* = 18.9892 (16) Å
*b* = 6.7660 (5) Å
*c* = 13.0072 (10) Å
β = 95.958 (3)°
*V* = 1662.2 (2) Å^3^
*Z* = 4
Mo *K*α radiation
μ = 0.08 mm^−1^
*T* = 296 K
0.35 × 0.28 × 0.25 mm

#### Data collection

Bruker Kappa APEXII CCD diffractometer
Absorption correction: multi-scan (*SADABS*; Bruker, 2005 ▶) *T*_min_ = 0.975, *T*_max_ = 0.985
8977 measured reflections
3006 independent reflections
2074 reflections with *I* > 2σ(*I*)
*R*_int_ = 0.028

#### Refinement

*R*[*F* ^2^ > 2σ(*F* ^2^)] = 0.051
*wR*(*F* ^2^) = 0.158
*S* = 1.05
3006 reflections
201 parameters
H-atom parameters constrained
Δρ_max_ = 0.30 e Å^−3^
Δρ_min_ = −0.17 e Å^−3^

### 

Data collection: *APEX2* (Bruker, 2009 ▶); cell refinement: *SAINT* (Bruker, 2009 ▶); data reduction: *SAINT*; program(s) used to solve structure: *SHELXS97* (Sheldrick, 2008 ▶); program(s) used to refine structure: *SHELXL97* (Sheldrick, 2008 ▶); molecular graphics: *ORTEP-3* (Farrugia, 1997 ▶) and *PLATON* (Spek, 2009 ▶); software used to prepare material for publication: *WinGX* (Farrugia, 1999 ▶) and *PLATON*.

## Supplementary Material

Crystal structure: contains datablock(s) global, I. DOI: 10.1107/S1600536812033594/hb6914sup1.cif

Structure factors: contains datablock(s) I. DOI: 10.1107/S1600536812033594/hb6914Isup2.hkl

Additional supplementary materials:  crystallographic information; 3D view; checkCIF report

## Figures and Tables

**Table 1:** Hydrogen-bond geometry (Å, °)

*D*—H⋯*A*	*D*—H	H⋯*A*	*D*⋯*A*	*D*—H⋯*A*
O1—H1⋯N1	0.82	1.86	2.593 (2)	148

**Fig. 1.**
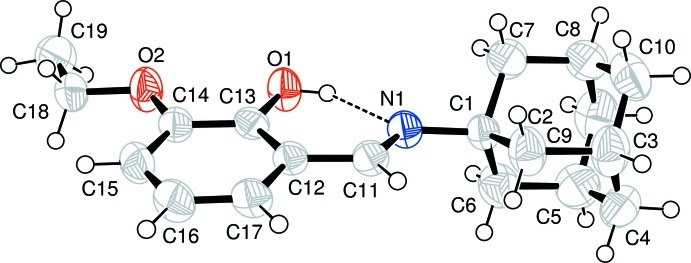
View of the title compound with displacement ellipsoids drawn at the 50% probability level. The dotted line represents the intramolecular hydrogen bond.

## supplementary crystallographic information

### Comment

The title compound (Fig. 1) has been synthesized as a derivative for the complexation and other studies.

The crystal structures of *N*-(1-adamantyl)salicylaldamine (Fernandez *et al.* 2001) has been published which is related to the title compound.

In the title compound the 3-adamantylamine the bond lengths and bond angles have normal values. The 3-ethoxy-2-hydroxybenzaldehyde group is planar with r.m.s. deviation of 0.0292 Å. There exist strong intramolecular H-bonding of O—H···N type (Table 1, Fig. 1) and *S*(6) ring motif is formed. There does not exist any intermolecular H-bonding.

### Experimental

Equimolar quantities of 3-adamantylamine and 3-ethoxy-2-hydroxybenzaldehyde were refluxed in ethanol for 2 h and yellow prisms of (I) were obtained after 72 h by the slow evaporation of the solvent at room temperature.

### Refinement

The H-atoms were positioned geometrically at C—H = 0.93–0.97 and O—H = 0.82 Å, respectively and included in the refinement as riding with *U*_iso_(H) = *xU*_eq_(C, O), where *x* = 1.5 for hydroxy & methyl and *x* = 1.2 for all other H-atoms.

### Crystal data

**Table:**

C_19_H_25_NO_2_	*F*(000) = 648
*M**_r_* = 299.40	*D*_x_ = 1.196 Mg m^−^^3^
Monoclinic, *P*2_1_/*c*	Mo *K*α radiation, λ = 0.71073 Å
Hall symbol: -P 2ybc	Cell parameters from 2074 reflections
*a* = 18.9892 (16) Å	θ = 2.2–25.3°
*b* = 6.7660 (5) Å	µ = 0.08 mm^−^^1^
*c* = 13.0072 (10) Å	*T* = 296 K
β = 95.958 (3)°	Prism, yellow
*V* = 1662.2 (2) Å^3^	0.35 × 0.28 × 0.25 mm
*Z* = 4	

### Data collection

**Table:**

Bruker Kappa APEXII CCD diffractometer	3006 independent reflections
Radiation source: fine-focus sealed tube	2074 reflections with *I* > 2σ(*I*)
Graphite monochromator	*R*_int_ = 0.028
Detector resolution: 8.10 pixels mm^-1^	θ_max_ = 25.3°, θ_min_ = 2.2°
ω scans	*h* = −22→22
Absorption correction: multi-scan (*SADABS*; Bruker, 2005)	*k* = −8→7
*T*_min_ = 0.975, *T*_max_ = 0.985	*l* = −15→14
8977 measured reflections	

### Refinement

**Table:**

Refinement on *F*^2^	Primary atom site location: structure-invariant direct methods
Least-squares matrix: full	Secondary atom site location: difference Fourier map
*R*[*F*^2^ > 2σ(*F*^2^)] = 0.051	Hydrogen site location: inferred from neighbouring sites
*wR*(*F*^2^) = 0.158	H-atom parameters constrained
*S* = 1.05	*w* = 1/[σ^2^(*F*_o_^2^) + (0.0682*P*)^2^ + 0.6627*P*] where *P* = (*F*_o_^2^ + 2*F*_c_^2^)/3
3006 reflections	(Δ/σ)_max_ < 0.001
201 parameters	Δρ_max_ = 0.30 e Å^−^^3^
0 restraints	Δρ_min_ = −0.17 e Å^−^^3^

### Special details

**Table:**

Geometry. Bond distances, angles *etc*. have been calculated using the rounded fractional coordinates. All su's are estimated from the variances of the (full) variance-covariance matrix. The cell e.s.d.'s are taken into account in the estimation of distances, angles and torsion angles
Refinement. Refinement of *F*^2^ against ALL reflections. The weighted *R*-factor *wR* and goodness of fit *S* are based on *F*^2^, conventional *R*-factors *R* are based on *F*, with *F* set to zero for negative *F*^2^. The threshold expression of *F*^2^ > σ(*F*^2^) is used only for calculating *R*-factors(gt) *etc*. and is not relevant to the choice of reflections for refinement. *R*-factors based on *F*^2^ are statistically about twice as large as those based on *F*, and *R*- factors based on ALL data will be even larger.

### Fractional atomic coordinates and isotropic or equivalent isotropic displacement parameters (Å^2^)

**Table:**

	*x*	*y*	*z*	*U*_iso_*/*U*_eq_	
O1	0.18741 (9)	−0.1124 (2)	0.42066 (12)	0.0557 (6)	
O2	0.10146 (9)	−0.0658 (2)	0.56419 (12)	0.0616 (6)	
N1	0.25760 (9)	0.0401 (3)	0.27943 (13)	0.0460 (6)	
C1	0.30814 (11)	0.0164 (3)	0.20220 (15)	0.0426 (7)	
C2	0.30683 (15)	0.1799 (4)	0.12148 (19)	0.0643 (9)	
C3	0.36170 (16)	0.1346 (4)	0.0448 (2)	0.0716 (10)	
C4	0.43359 (16)	0.1258 (5)	0.1053 (3)	0.0863 (12)	
C5	0.43686 (15)	−0.0407 (5)	0.1834 (2)	0.0802 (11)	
C6	0.38174 (13)	0.0023 (5)	0.2599 (2)	0.0710 (10)	
C7	0.29071 (14)	−0.1797 (4)	0.1462 (2)	0.0646 (9)	
C8	0.34638 (15)	−0.2240 (4)	0.0709 (2)	0.0688 (10)	
C9	0.41789 (16)	−0.2344 (4)	0.1310 (3)	0.0820 (11)	
C10	0.34335 (16)	−0.0602 (4)	−0.0073 (2)	0.0746 (10)	
C11	0.22809 (11)	0.2044 (3)	0.29534 (16)	0.0445 (7)	
C12	0.18045 (11)	0.2304 (3)	0.37448 (15)	0.0408 (6)	
C13	0.16242 (11)	0.0702 (3)	0.43468 (16)	0.0415 (7)	
C14	0.11691 (11)	0.0998 (3)	0.51114 (16)	0.0453 (7)	
C15	0.09234 (12)	0.2874 (3)	0.52875 (18)	0.0551 (8)	
C16	0.11149 (13)	0.4469 (3)	0.47037 (19)	0.0580 (8)	
C17	0.15451 (12)	0.4186 (3)	0.39414 (17)	0.0497 (7)	
C18	0.05855 (13)	−0.0432 (4)	0.64640 (18)	0.0596 (8)	
C19	0.05370 (18)	−0.2399 (4)	0.6973 (2)	0.0825 (11)	
H1	0.21393	−0.11002	0.37479	0.0835*	
H2A	0.31785	0.30560	0.15522	0.0772*	
H2B	0.25991	0.18906	0.08441	0.0772*	
H3	0.36090	0.23963	−0.00722	0.0857*	
H4A	0.44339	0.25035	0.14086	0.1035*	
H4B	0.46944	0.10551	0.05839	0.1035*	
H5	0.48437	−0.04838	0.22057	0.0964*	
H6A	0.38278	−0.10251	0.31092	0.0851*	
H6B	0.39340	0.12553	0.29582	0.0851*	
H7A	0.24405	−0.17209	0.10823	0.0775*	
H7B	0.29033	−0.28569	0.19639	0.0775*	
H8	0.33547	−0.35033	0.03600	0.0825*	
H9A	0.45306	−0.26720	0.08468	0.0984*	
H9B	0.41810	−0.33803	0.18250	0.0984*	
H10A	0.29614	−0.05317	−0.04361	0.0895*	
H10B	0.37638	−0.08758	−0.05763	0.0895*	
H11	0.23744	0.31231	0.25458	0.0534*	
H15	0.06262	0.30738	0.58030	0.0662*	
H16	0.09494	0.57293	0.48333	0.0695*	
H17	0.16675	0.52549	0.35474	0.0597*	
H18A	0.01173	0.00252	0.61999	0.0716*	
H18B	0.07941	0.05308	0.69578	0.0716*	
H19A	0.10031	−0.29447	0.71201	0.1236*	
H19B	0.02528	−0.32722	0.65201	0.1236*	
H19C	0.03238	−0.22430	0.76057	0.1236*	

### Atomic displacement parameters (Å^2^)

**Table:**

	*U*^11^	*U*^22^	*U*^33^	*U*^12^	*U*^13^	*U*^23^
O1	0.0775 (12)	0.0295 (8)	0.0652 (10)	0.0063 (7)	0.0323 (8)	0.0032 (7)
O2	0.0801 (12)	0.0453 (9)	0.0655 (10)	−0.0012 (8)	0.0373 (9)	0.0021 (8)
N1	0.0572 (11)	0.0347 (10)	0.0476 (10)	0.0018 (8)	0.0123 (9)	0.0019 (8)
C1	0.0505 (12)	0.0363 (11)	0.0421 (11)	0.0029 (9)	0.0099 (9)	0.0011 (9)
C2	0.0833 (18)	0.0520 (14)	0.0615 (15)	0.0105 (13)	0.0257 (13)	0.0061 (12)
C3	0.093 (2)	0.0592 (17)	0.0673 (16)	0.0058 (14)	0.0314 (15)	0.0138 (14)
C4	0.082 (2)	0.081 (2)	0.103 (2)	−0.0267 (17)	0.0432 (18)	−0.0229 (19)
C5	0.0530 (16)	0.104 (2)	0.0823 (19)	0.0053 (16)	0.0004 (14)	−0.0090 (19)
C6	0.0649 (16)	0.088 (2)	0.0594 (15)	0.0051 (15)	0.0026 (13)	−0.0030 (14)
C7	0.0686 (16)	0.0498 (14)	0.0779 (17)	−0.0026 (12)	0.0194 (14)	−0.0106 (13)
C8	0.0762 (18)	0.0523 (15)	0.0799 (18)	0.0004 (13)	0.0184 (15)	−0.0162 (14)
C9	0.0751 (19)	0.075 (2)	0.099 (2)	0.0268 (16)	0.0233 (17)	0.0066 (17)
C10	0.0787 (18)	0.087 (2)	0.0590 (15)	0.0130 (16)	0.0114 (14)	−0.0144 (15)
C11	0.0547 (13)	0.0335 (11)	0.0460 (12)	−0.0028 (10)	0.0087 (10)	0.0035 (9)
C12	0.0473 (12)	0.0320 (10)	0.0435 (11)	−0.0008 (9)	0.0062 (9)	−0.0002 (9)
C13	0.0476 (12)	0.0297 (10)	0.0472 (12)	0.0010 (9)	0.0053 (9)	−0.0039 (9)
C14	0.0510 (13)	0.0377 (12)	0.0485 (12)	−0.0017 (10)	0.0112 (10)	−0.0014 (10)
C15	0.0586 (14)	0.0498 (14)	0.0596 (14)	0.0061 (11)	0.0185 (11)	−0.0095 (12)
C16	0.0674 (15)	0.0353 (12)	0.0726 (16)	0.0121 (11)	0.0140 (13)	−0.0067 (11)
C17	0.0615 (14)	0.0308 (11)	0.0574 (13)	0.0039 (10)	0.0084 (11)	0.0023 (10)
C18	0.0660 (15)	0.0659 (16)	0.0503 (13)	−0.0106 (12)	0.0216 (12)	−0.0087 (12)
C19	0.108 (2)	0.081 (2)	0.0640 (17)	−0.0329 (17)	0.0350 (16)	−0.0010 (15)

### Geometric parameters (Å, º)

**Table:**

O1—C13	1.343 (2)	C18—C19	1.494 (4)
O2—C14	1.364 (3)	C2—H2A	0.9700
O2—C18	1.418 (3)	C2—H2B	0.9700
O1—H1	0.8200	C3—H3	0.9800
N1—C11	1.272 (3)	C4—H4A	0.9700
N1—C1	1.468 (3)	C4—H4B	0.9700
C1—C2	1.524 (3)	C5—H5	0.9800
C1—C6	1.519 (3)	C6—H6A	0.9700
C1—C7	1.533 (3)	C6—H6B	0.9700
C2—C3	1.546 (4)	C7—H7A	0.9700
C3—C4	1.505 (4)	C7—H7B	0.9700
C3—C10	1.506 (4)	C8—H8	0.9800
C4—C5	1.514 (5)	C9—H9A	0.9700
C5—C9	1.504 (4)	C9—H9B	0.9700
C5—C6	1.545 (4)	C10—H10A	0.9700
C7—C8	1.543 (4)	C10—H10B	0.9700
C8—C10	1.501 (4)	C11—H11	0.9300
C8—C9	1.497 (4)	C15—H15	0.9300
C11—C12	1.450 (3)	C16—H16	0.9300
C12—C17	1.398 (3)	C17—H17	0.9300
C12—C13	1.400 (3)	C18—H18A	0.9700
C13—C14	1.398 (3)	C18—H18B	0.9700
C14—C15	1.380 (3)	C19—H19A	0.9600
C15—C16	1.390 (3)	C19—H19B	0.9600
C16—C17	1.362 (3)	C19—H19C	0.9600
			
C14—O2—C18	117.61 (17)	C5—C4—H4A	110.00
C13—O1—H1	109.00	C5—C4—H4B	110.00
C1—N1—C11	122.50 (19)	H4A—C4—H4B	108.00
N1—C1—C2	115.28 (19)	C4—C5—H5	110.00
N1—C1—C7	107.18 (18)	C6—C5—H5	110.00
C2—C1—C6	109.5 (2)	C9—C5—H5	110.00
N1—C1—C6	107.58 (17)	C1—C6—H6A	110.00
C6—C1—C7	108.8 (2)	C1—C6—H6B	110.00
C2—C1—C7	108.28 (18)	C5—C6—H6A	110.00
C1—C2—C3	109.7 (2)	C5—C6—H6B	110.00
C2—C3—C10	108.9 (2)	H6A—C6—H6B	108.00
C4—C3—C10	110.8 (2)	C1—C7—H7A	110.00
C2—C3—C4	107.8 (2)	C1—C7—H7B	110.00
C3—C4—C5	110.6 (3)	C8—C7—H7A	110.00
C4—C5—C6	108.0 (3)	C8—C7—H7B	110.00
C4—C5—C9	110.7 (3)	H7A—C7—H7B	108.00
C6—C5—C9	108.0 (3)	C7—C8—H8	110.00
C1—C6—C5	110.1 (2)	C9—C8—H8	110.00
C1—C7—C8	110.0 (2)	C10—C8—H8	110.00
C7—C8—C9	108.8 (2)	C5—C9—H9A	109.00
C9—C8—C10	111.0 (2)	C5—C9—H9B	109.00
C7—C8—C10	107.8 (2)	C8—C9—H9A	109.00
C5—C9—C8	111.0 (2)	C8—C9—H9B	109.00
C3—C10—C8	110.5 (2)	H9A—C9—H9B	108.00
N1—C11—C12	122.66 (19)	C3—C10—H10A	110.00
C11—C12—C17	119.86 (19)	C3—C10—H10B	110.00
C13—C12—C17	119.43 (19)	C8—C10—H10A	110.00
C11—C12—C13	120.66 (18)	C8—C10—H10B	110.00
O1—C13—C14	118.80 (18)	H10A—C10—H10B	108.00
C12—C13—C14	119.47 (19)	N1—C11—H11	119.00
O1—C13—C12	121.73 (19)	C12—C11—H11	119.00
O2—C14—C15	125.29 (19)	C14—C15—H15	120.00
C13—C14—C15	119.64 (19)	C16—C15—H15	120.00
O2—C14—C13	115.06 (18)	C15—C16—H16	120.00
C14—C15—C16	120.8 (2)	C17—C16—H16	120.00
C15—C16—C17	120.0 (2)	C12—C17—H17	120.00
C12—C17—C16	120.69 (19)	C16—C17—H17	120.00
O2—C18—C19	107.8 (2)	O2—C18—H18A	110.00
C1—C2—H2A	110.00	O2—C18—H18B	110.00
C1—C2—H2B	110.00	C19—C18—H18A	110.00
C3—C2—H2A	110.00	C19—C18—H18B	110.00
C3—C2—H2B	110.00	H18A—C18—H18B	108.00
H2A—C2—H2B	108.00	C18—C19—H19A	109.00
C2—C3—H3	110.00	C18—C19—H19B	109.00
C4—C3—H3	110.00	C18—C19—H19C	109.00
C10—C3—H3	110.00	H19A—C19—H19B	109.00
C3—C4—H4A	109.00	H19A—C19—H19C	109.00
C3—C4—H4B	110.00	H19B—C19—H19C	109.00
			
C18—O2—C14—C13	176.74 (19)	C9—C5—C6—C1	−60.4 (3)
C18—O2—C14—C15	−2.2 (3)	C4—C5—C9—C8	−56.4 (3)
C14—O2—C18—C19	−175.7 (2)	C6—C5—C9—C8	61.6 (3)
C11—N1—C1—C2	−17.3 (3)	C1—C7—C8—C9	59.1 (3)
C11—N1—C1—C6	105.2 (2)	C1—C7—C8—C10	−61.3 (3)
C11—N1—C1—C7	−137.9 (2)	C7—C8—C9—C5	−61.3 (3)
C1—N1—C11—C12	−177.34 (18)	C10—C8—C9—C5	57.1 (3)
N1—C1—C2—C3	−179.26 (19)	C7—C8—C10—C3	61.8 (3)
C6—C1—C2—C3	59.3 (3)	C9—C8—C10—C3	−57.2 (3)
C7—C1—C2—C3	−59.3 (3)	N1—C11—C12—C13	−2.8 (3)
N1—C1—C6—C5	175.2 (2)	N1—C11—C12—C17	174.5 (2)
C2—C1—C6—C5	−58.8 (3)	C11—C12—C13—O1	−1.0 (3)
C7—C1—C6—C5	59.4 (3)	C11—C12—C13—C14	179.38 (19)
N1—C1—C7—C8	−174.59 (19)	C17—C12—C13—O1	−178.3 (2)
C2—C1—C7—C8	60.5 (3)	C17—C12—C13—C14	2.1 (3)
C6—C1—C7—C8	−58.5 (2)	C11—C12—C17—C16	−177.9 (2)
C1—C2—C3—C4	−60.5 (3)	C13—C12—C17—C16	−0.6 (3)
C1—C2—C3—C10	59.8 (3)	O1—C13—C14—O2	−0.9 (3)
C2—C3—C4—C5	62.6 (3)	O1—C13—C14—C15	178.1 (2)
C10—C3—C4—C5	−56.5 (3)	C12—C13—C14—O2	178.77 (18)
C2—C3—C10—C8	−61.5 (3)	C12—C13—C14—C15	−2.3 (3)
C4—C3—C10—C8	57.0 (3)	O2—C14—C15—C16	179.8 (2)
C3—C4—C5—C6	−62.0 (3)	C13—C14—C15—C16	0.9 (3)
C3—C4—C5—C9	56.1 (3)	C14—C15—C16—C17	0.6 (4)
C4—C5—C6—C1	59.3 (3)	C15—C16—C17—C12	−0.7 (4)

### Hydrogen-bond geometry (Å, º)

**Table:**

*D*—H···*A*	*D*—H	H···*A*	*D*···*A*	*D*—H···*A*
O1—H1···N1	0.82	1.86	2.593 (2)	148
